# Electrocorticographic description of the effects of anticonvulsant drugs used to treat lidocaine‐induced seizures

**DOI:** 10.1002/brb3.1940

**Published:** 2020-12-25

**Authors:** George Francisco S. Santos, Luan Oliveira Ferreira, Bruna Gerrits Mattos, Eliniete J. Fidelis, Alisson S. de Souza, Paula S. Batista, Cecilia A. F. Manoel, Diego Arthur C. Cabral, Vanessa Jóia de Mello, Dielly Catrina Favacho Lopes, Moisés Hamoy

**Affiliations:** ^1^ Laboratory of the Pharmacology and Toxicology of Natural Products Institute of Biological Sciences Federal University of Pará Belém Brazil; ^2^ Laboratory of Experimental Neuropathology João de Barros Barreto University Hospital Federal University of Pará Belém Brazil

**Keywords:** electrocorticography, electromyography, epilepsy, lidocaine, seizure

## Abstract

**Introduction:**

Local anesthetics are widely used in clinical practice. While toxicity is rare, these drugs can cause potentially lethal seizures.

**Objective:**

In the present study, we investigated the electrocorticographic (ECoG) and electromyographic patterns of seizures induced by acute lidocaine (LA) toxicity and treated with anticonvulsant drugs. The study used adult male Wistar rats to describe of the seizure‐related behavior of LA and investigated the treatment with anticonvulsant drugs.

**Results:**

The use of LA resulted in clear changes in the ECoG pattern, which presented characteristics of *Status epilepticus*, with increased intensity in all brainwaves. The decomposition of the cerebral waves showed an increase in the beta and gamma waves that may be related to tonic–clonic seizure. Although the treatment with anticonvulsants drugs reduces the power of brainwaves at frequencies between 1 and 40 Hz compared to the LA group, but only diazepam (DZP) was able to decrease the intensity of oscillations. The muscle contraction power also indicated a difference in the effectiveness of the three treatments.

**Conclusion:**

The sum of the evidence indicates that LA causes *status epilepticus* and that DZP is the most effective treatment for the control of these seizures, by restoring the systemic values to levels close to those recorded in the control group.

## Graphical abstract

Lidocaine (LA) intraperitoneally resulted in changes in the electrocorticographic pattern, with characteristics of Status epilepticus. Treatment with diazepam reduces the power of brainwaves at frequencies between 1 and 40 Hz compared to the LA group.

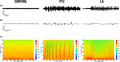

## 1 INTRODUCTION

Lidocaine (LA) is an amide‐type local anesthetic and antiarrhythmic drug, which has been marketed since the 1940 (McBride et al., 2017). Excessive doses can lead to adverse effects on the cardiovascular system, such as arrhythmia, and on the central nervous system (CNS), including unconsciousness, *status epilepticus* or coma. Its main mechanism of action is through the blocking of cell signaling by hindering the influx of sodium ions via the Na^+^‐channels, and possibly by increasing the depolarization threshold in the nociceptive pathways (Butterworth & Strichartz, 1990; Hancı et al., 2013; McBride et al., 2017). Acute LA toxicity is dosage‐dependent and is related directly to the blood concentration (Alster, 2013; Ayas & İsik, 2014). Although systemic toxicity from local anesthetics is rare, they can be potentially lethal by causing seizures, arrhythmias, and cardiovascular collapse (Hasan et al., 2017). Miscalculation of the dose, injection of the drug into a blood vessel, and the repeated administration of therapeutic doses are the principal causes of systemic toxicity (Ciechanowicz & Patil, 2012). Acute LA toxicity is diagnosed based on the clinical symptoms and/or plasma levels. Potential alternative diagnoses include altered level of consciousness, epilepsy, and crack and cocaine toxicity (Neal et al., 2018; Sekimoto et al., 2017).

Lidocaine has two different effects on the CNS in animal models, depending on the dosage administered. At doses of below 40 mg/kg, LA can induce neuroprotection in an ischemic stroke or spinal cord injury (Apaydin & Büket, 2001; Lei et al., 2001). At a toxic dose (over 50 mg/kg), however, LA may cause seizures (Sawaki et al., 2000). The neurotoxicity induced by this local anesthetic is based on the depression of inhibitory neurons, which potentializes excitatory neuronal activity (de Jong et al., 1969). This may occur through the inhibition of the release of gamma‐aminobutyric acid (GABA; Nakahata et al., 2010). Anticonvulsant drugs, such as benzodiazepines and barbiturates, have been shown to decrease seizures induced by local anesthetics administered systemically (Torp & Simon, 2019).

The present study describes the behavioral responses of male Wistar rats exposed to acute LA toxicity, together with their electrocorticographic (ECoG) and electromyographic (EMG) alterations. The effectiveness of three different anticonvulsant drugs for the attenuation of LA‐induced seizures was also assessed.

## 2 MATERIALS AND METHODS

### 2.1 Animals

Ninety‐nine adult male Wistar rats (280 ± 40g, aged 10–12 weeks) were obtained from the Central Animal Facility of the Federal University of Pará. All the animals were housed in standard white cages (48 cm × 38 cm × 21 cm) under controlled conditions (22 ± 2°C; 12/12 hr light/dark cycle) with ad libitum access to food and water. All experimental procedures were conducted in accordance with the principles of laboratory animal care (National Research Council, 2011) and were approved by the Ethics Committee on Experiments in Animals of the Federal University of Pará (CEUA no. 6907241117). All necessary precautions were taken to prevent animal suffering and distress.

### 2.2 Drugs

Ketamine was purchased from König and xylazine from Vallée, while the LA was obtained from Hipolabor. The convulsing agent pentylenetetrazole (PTZ) was purchased from Sigma‐Aldrich. The anticonvulsant compounds were obtained from two different sources, with phenobarbital (PBT) being purchased from Aventis‐Pharma, and phenytoin (PHT) and diazepam (DZP) from União Química.

### 2.3 Experimental design

The study was had two phases. Phase 1 consisted of the description of the seizure‐related behavior of the LA group (*n* = 9 animals) that received a 60 mg/kg dose of LA, together with ECoG recordings. This LA dosage was chosen based on the results of Blas‐Valdivia et al. (2007). In phase 2, the rats were divided randomly into a vehicle‐treated (control), PTZ (positive control of seizure induced, 60 mg/kg), and LA groups (*n* = 9 animals per group). In phase 2, 10 min after the induction of seizures by LA, the rats were treated with three anticonvulsant drugs (*n* = 9 animals per group), as used by Hamoy et al. (2018): (a) DZP (10 mg/kg), (b) PBT (10 mg/kg), or (c) PHT (10 mg/kg). In all cases, ECoG or EMG recordings were obtained. All drugs were administered via intraperitoneal (i.p.) injection. The vehicle group received a 0.9% physiological solution at an equivalent volume (ml) to weight (kg) ratio.

### 2.4 Description of the seizure‐related behavior

Seizure‐related behavior was observed after the injection of LA. The latency of the seizures was recorded, and the behavioral modifications were classified in seven clinically identifiable stages, as proposed by Hamoy et al. (2018): (a) akinesia and motionless staring; (b) rapid whisker movements; (c) tremors of the forelimbs; (d) tail stiffening; (e) lack of coordination and generalized tremors; (f) impairment of the postural reflex; and (g) tonic–clonic seizures.

### 2.5 Electrocorticographic recordings and data analyses

The ECoG recordings were obtained using the procedures described by Estumano et al. (2019). For this, the animals were anesthetized with ketamine hydrochloride (80 mg/kg, i.p.) and xylazine hydrochloride (10 mg/kg, i.p.), and after the abolishment of their corneal reflex, they were placed in a stereotaxic apparatus. The skull was exposed, and stainless‐steel electrodes (tip exposure, 1.0 mm in diameter) were placed on the dura mater above the frontal cortex at the coordinates of the bregma—0.96 ± 1.0 mm lateral. Five days after surgery, the ECoG was recorded using a digital data acquisition system and the tracing was registered in mV (millivolts), and the offline analysis was run as described by Hamoy et al. (2018).

The recordings were obtained following the standard protocol: The animals were immobilized carefully for 10 min to allow for accommodation and avoid possible interference with the recording. Basal ECoG activity was recorded for 30 min, which was used as the control treatment in the ECoG analyses. The LA or PTZ was then administered, and the ECoG activity was recorded for a further 30 min. As LA seizures can last for many hours, the animals were euthanized at this point to avoid further distress.

The animals were treated following a similar protocol for the evaluation of the seizures induced by LA and controlled by anticonvulsants. Fort this, the animals received one of the anticonvulsant agents (DZP, PBT, or PHT) 10 min after the LA injection. After the application of the anticonvulsant drugs, the ECoG was recorded for 30 min.

The analyses were run at a frequency of up to 50 Hz, split into the delta (1–4 Hz), theta (4–8 Hz), alpha (8–12 Hz), beta (12–28 Hz), and gamma (28–40 Hz) bands (Jalilifar et al., 2017) for the interpretation of the dynamics of the development of the seizure Hamoy et al. (2018).

### 2.6 Electromyographic recordings and data analyses

To demonstrate muscle activity during LA‐induced seizure, the electrodes were implanted in the masseter muscle in parallel 5 mm above their fixation in the jaw. The recording was amplified 2000×, and the data were processed as in the electrocorticography.

### 2.7 Statistical analysis

The data were analyzed in GraphPad Prism, version 8 (GraphPad Software Inc.), and *p* values of less than .05 were considered to be statistically significant. The data are presented as means ± *SD*, and the *F* and *p* values are included, where pertinent. Between‐group comparisons were based on a one‐way ANOVA followed by Tukey's test for multiple comparisons.

## 3 RESULTS

### 3.1 Seizure‐related behavior induced by LA

Seizure‐related behavior was observed following the injection of LA at a dose of 60 mg/kg. The animals presented continuous and progressive stages of seizure. The mean latency of the first behavioral change, akinesia and motionless staring, was 99.7 ± 40.4 s, followed by rapid whisker movements (stage 2) at a mean of 101.7 ± 39.3 s. Tremors of the forelimbs (stage 3) appeared at 143.9 ± 85.7 s, and tail stiffening (stage 4) at 232.0 ± 159.2 s. Progression to the critical stages began with a lack of coordination and generalized tremors (stage 5) at 232.9 ± 86.6 s, until the appearance of tonic–clonic seizures (stage 7) at 544.4 ± 127.5 s.

### 3.2 Acute LA toxicity alters brainwave patterns

**FIGURE 1:**
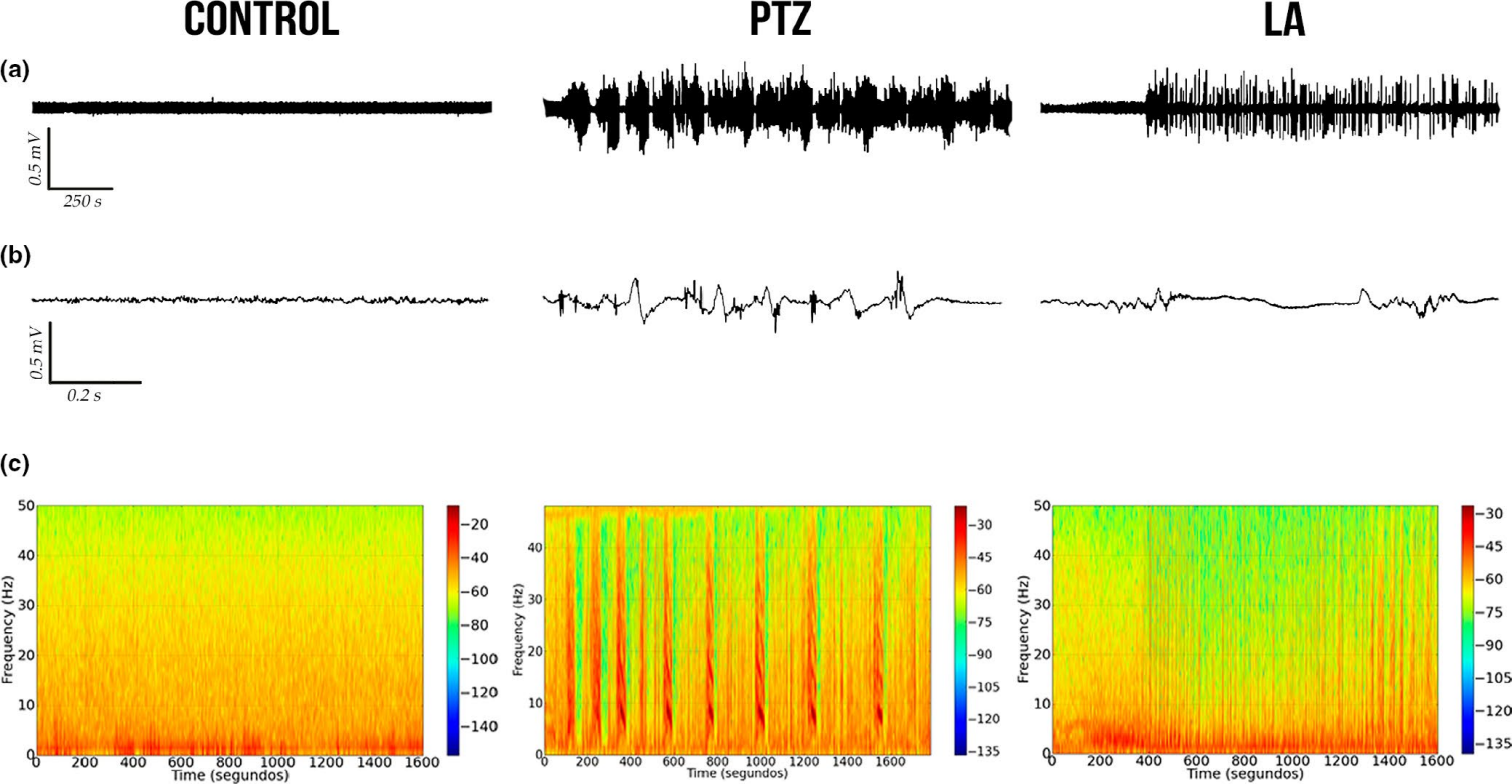
Electrocorticographic recordings of the control and seizure‐induced animals performed for 30 min. (a) Record of linear tracing performed by electrocorticographic (ECoG). (b) Representative fragment of 1 s from ECoG tracing. (c) Frequency spectrogram. Control group (left), PTZ group (center), and LA group (right). LA, lidocaine; mV, millivolts; PTZ, pentylenetetrazole

The ECoG recordings of the control groups were of low amplitude (typically below 0.1 mV) with the highest energy concentrated below 10 Hz (Figure 1a). In the PTZ group, by contrast, ECoG changes can be observed, with cyclic peaks that reached amplitudes of over 0.3 mV, characteristic of seizures (Figure 1b). The LA group presented a pattern that is characteristic of a convulsive state, but with variations in amplitude distinct from those observed in the PTZ group (Figure 1c).

**FIGURE 2:**
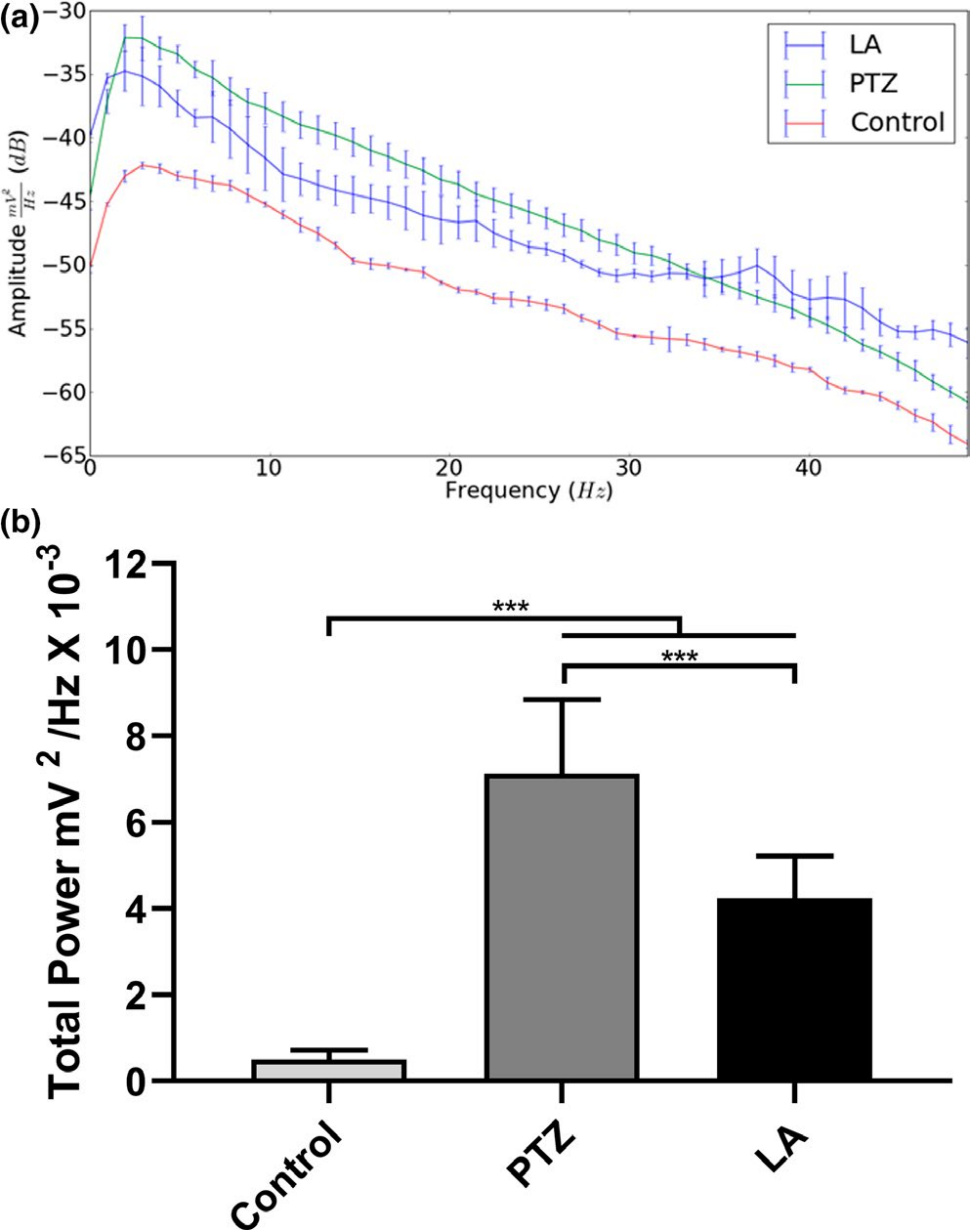
Linear frequency distribution representative of the brainwaves during seizure and total power quantitative recorded by electrocorticographic. (a) Representative distribution of the linear frequency in the groups, at frequencies of up to 50 Hz. (b) Quantitative distribution of the total linear power of the brainwaves. The data are expressed as the mean ± *SD*(*n* = 9 per group; ***p < .001). dB, decibel; LA, lidocaine; PTZ, pentylenetetrazole

The decomposition of the distribution of the spectral power revealed greater amplitude in all the brainwaves of the PTZ and LA groups in comparison with the control group (Figure 2a). Significant variation among the groups was found in the analysis of the distribution of the linear frequencies of up to 50 Hz (*F*_(2,24)_ = 74.71; *p* < .0001). In this case, both the PTZ and the LA groups had higher total spectral power than the control group; however, a significantly smaller increase was observed in the LA group in comparison with the PTZ group (control: 0.50 ± 0.23 mV^2^/Hz × 10^–3^; PTZ: 7.12 ± 1.72 mV^2^/Hz × 10^–3^; LA: 4.25 ± 0.98 mV^2^/Hz × 10^–3^; *p* < .001 for all comparisons; Figure 2b).

**FIGURE 3:**
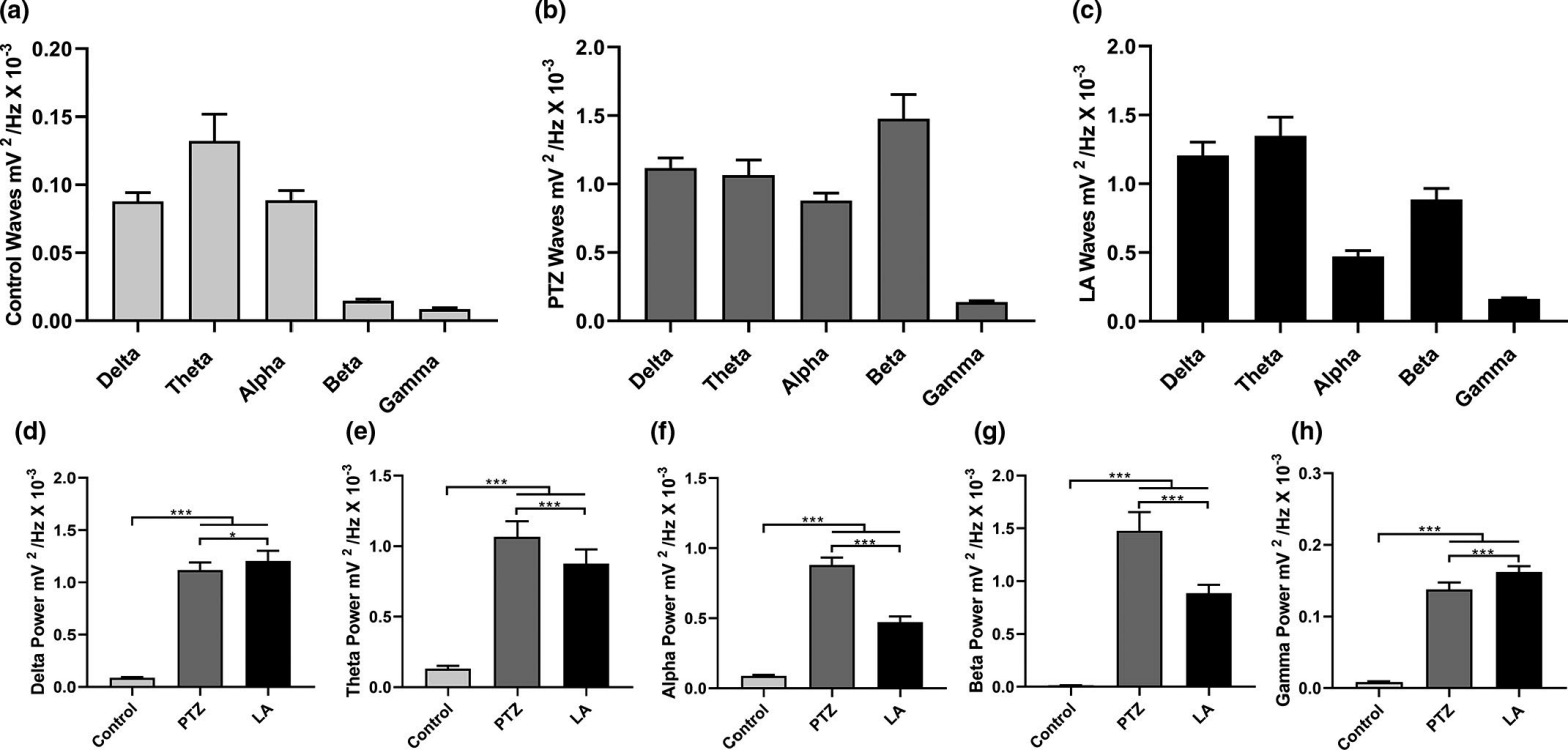
Quantitative decomposition of the brainwaves in the control animals and during seizures induced by pentylenetetrazole or lidocaine: (a) control; (b) pentylenetetrazole; and (c) lidocaine. Quantitative linear frequency distribution of the (d) delta waves; (e) theta waves; (f) alpha waves; (g) beta waves; and (h) gamma waves. The data are expressed as the mean ± *SD*(*n* = 9 per group; *p < .05 and ***p < .001). Hz, Hertz; LA, lidocaine; mV, millivolts; PTZ, pentylenetetrazole

The decomposition of the brainwaves was also analyzed, beginning with the power wave distribution in the control, PTZ, and LA groups. Difference can be noted between the wave amplitudes of the seizure‐inducing agents, which indicate that the drugs could represent different kind of seizure (Figure 3a–c).

The delta waves varied significantly among treatments (*F*_(2,24)_ = 710.1; *p* < .0001), with a significant increase being found in the LA group (1.20 ± 0.10 mV^2^/Hz × 10^–3^) in comparison with the PTZ group (1.12 ± 0.07 mV^2^/Hz × 10^–3^; *p* = .0322). Both groups were also significantly different from the control group (0.09 ± 0.01 mV^2^/Hz x 10^–3^; *p* < .001; Figure 3d).

The theta wave frequencies also varied significantly among groups (*F*_(2,24)_ = 288.0; *p* < .0001). Animals from PTZ group had a higher mean theta power (1.07 ± 0.11 mV^2^/Hz × 10^–3^) than that those of the LA group (0.88 ± 0.10 mV^2^/Hz × 10^–3^; *p* = .0003), and both groups were significantly higher than the control group (0.13 ± 0.02 mV^2^/Hz × 10^–3^; *p* < .001; Figure 3e).

As in the other cases, the alpha waves also varied significantly among all the groups (*F*_(2,24)_ = 900.7; *p* < .0001), and once again, the control animals had the lowest alpha power of all the groups, with a mean of 0.09 ± 0.01 mV^2^/Hz × 10^–3^ (*p* < .001 for all comparisons). The PTZ‐induced seizures also presented significantly higher alpha wave power than the LA‐induced ones (PTZ: 0.88 ± 0.05 mV^2^/Hz × 10^–3^; LA: 0.47 ± 0.04 mV^2^/Hz × 10^–3^; *p* < .001; Figure 3f).

Once, all the groups varied significantly in their beta wave power (*F*_(2,24)_ = 394.7; *p* < .0001), with the PTZ group having a significantly higher mean beta power than the LA group (PTZ: 1.48 ± 0.17 mV^2^/Hz × 10^–3^; LA: 0.89 ± 0.08 mV^2^/Hz × 10^–3^; *p* < .001). Both groups were also significantly higher than the control group (0.014 ± 0.001 mV^2^/Hz × 10^–3^; *p* < .001; Figure 3g).

Similarly, gamma wave power also varied significantly among treatments (*F*_(2,24)_ = 1,133; *p* < .0001), with LA‐induced seizures presenting higher values than PTZ‐induced seizures, and both these groups being significantly different from the control group (control: 0.01 ± 0.001 mV^2^/Hz × 10^–3^; PTZ: 0.14 ± 0.01 mV^2^/Hz × 10^–3^; LA: 0.16 ± 0.01 mV^2^/Hz × 10^–3^; *p* < .001 for all comparisons; Figure 3h).

### 3.3 Lidocaine‐induced seizures alter ECoG readings and are attenuated by anticonvulsant drugs

**FIGURE 4:**
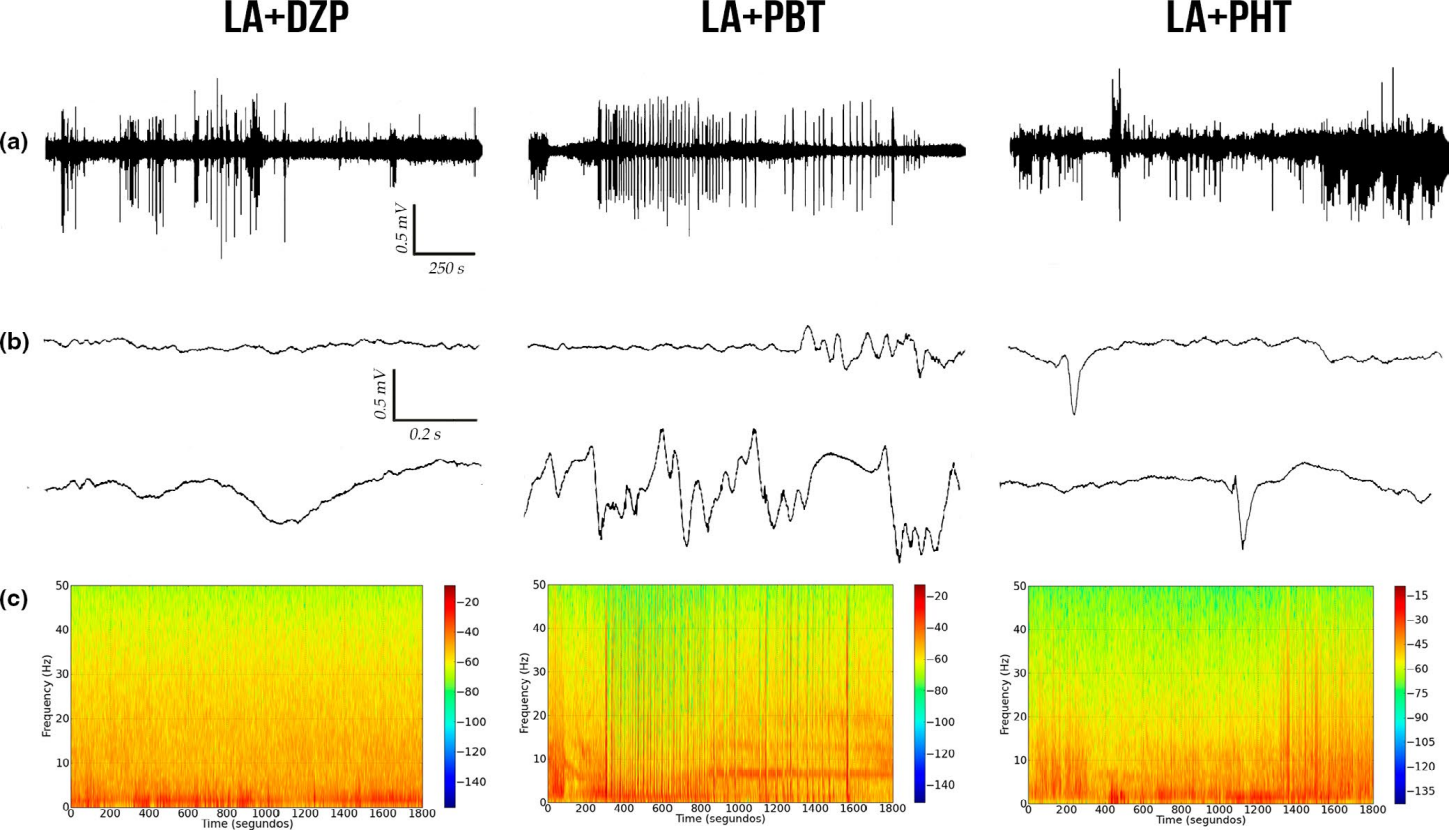
Eletrocorticographic recordings of lidocaine‐induced seizures treated with anticonvulsant drugs performed for 30 min: (a) Record of linear tracing performed by electrocorticographic (ECoG); (b) two representative fragment of two seconds from ECoG tracing; and (c) frequency spectrogram. LA‐induced seizure controlled by diazepam (left); LA‐induced seizure partially controlled by phenobarbital (center), and LA‐induced seizure not controlled by phenytoin (right). DZP, diazepam; LA, lidocaine; mV, millivolts; PBT, phenobarbital; PHT, phenytoin

After describing the LA‐induced seizures, the anticonvulsant drugs were tested to determine whether they are capable of reducing or abolishing the alterations in the brainwaves caused by the LA. For this, 10 min after the initiation of the LA‐induced seizures DZP, PBT, or PHT was administered, and the ECoG readings of the animals were recorded. This analysis showed that DZP decreased seizure activity after 15 min, with amplitude variation of up to 0.5 mV. During the second part of the recording, amplitude was reduced to 0.1 mV, with minimal variation in the tracing (Figure 4a).

By contrast, neither PBT nor PHT was able to abolish the peaks in the ECoG over the 30‐min recording period. The PBT presented cyclic variation, with peaks of amplitude of 0.5 mV, although the interval between the cycles did increase gradually (Figure 4b). However, PHT had the least capacity to control the peaks in amplitude, with both the amplitude and the intensity of the cycles being maintained throughout the whole recording session (Figure 4c).

**FIGURE 5:**
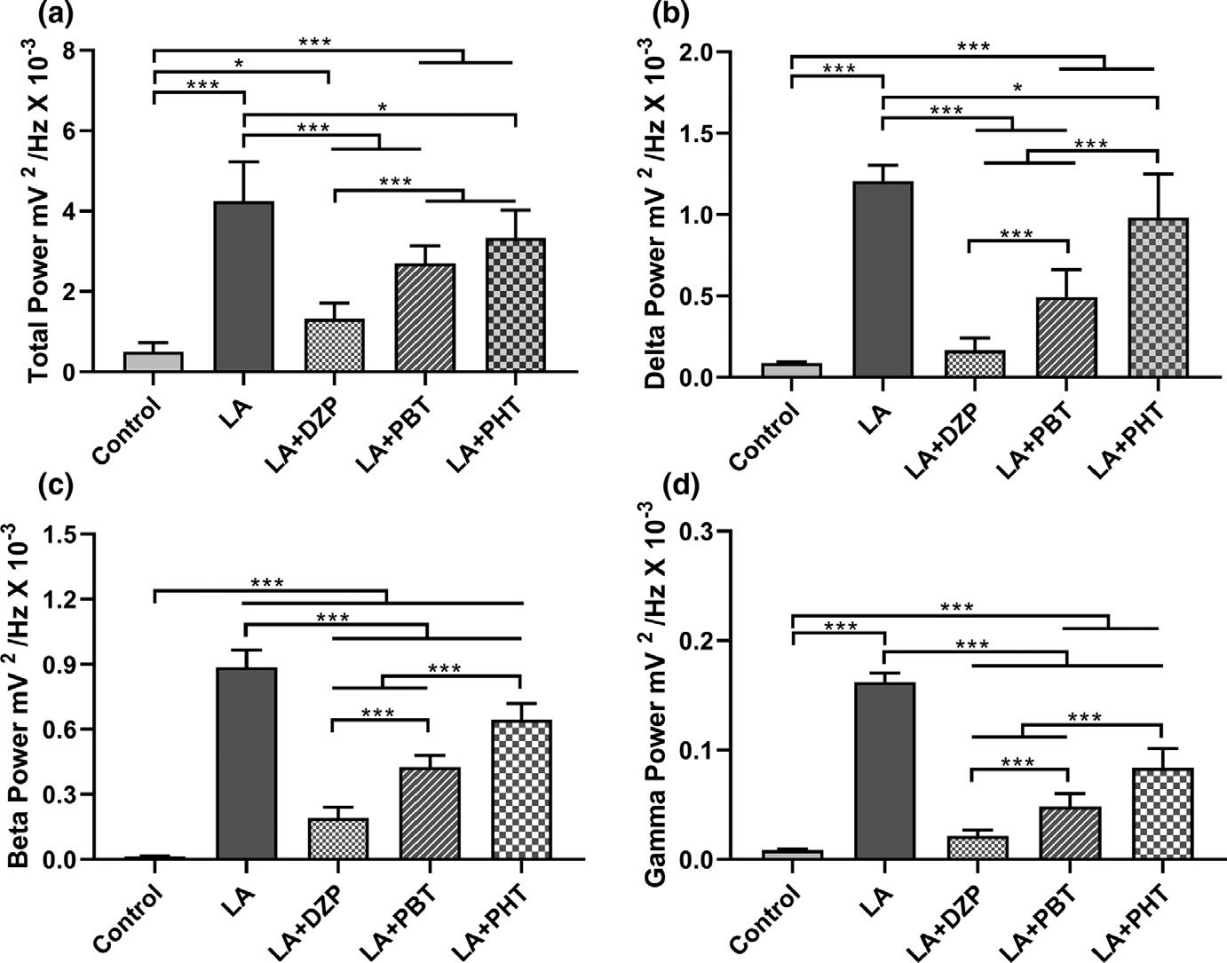
Quantitative total power and decomposition of the brainwaves in lidocaine‐induced seizures and those treated with anticonvulsant drugs: (a) Quantitative distribution of the total linear power of the brainwaves of the lidocaine‐induced seizures treated with anticonvulsant drugs. Quantitative linear frequency distribution of the (b) delta waves; (c) beta waves; and (d) gamma waves. The data are expressed as the mean ± *SD*(*n* = 9 per group; *p < .05 and ***p < .001). DZP, diazepam; Hz, Hertz; LA, lidocaine; mV, millivolts; PBT, phenobarbital; PHT, phenytoin

The decomposition of the total spectral power distribution revealed that there was a significant difference among the treatments (*F*_(4,40)_ = 56.41; *p* < .0001; Figure 5a). While all the treatments decreased the LA‐induced seizures (LA + DZP: 1.32 ± 0.40 mV^2^/Hz × 10^–3^ and LA + PTB: 2.71 ± 0.43 mV^2^/Hz × 10^–3^ vs. LA: 4.25 ± 0.98 mV^2^/Hz × 10^–3^; *p* < .001), PHT was less effective than DZP (LA + PHT: 3.34 ± 0.68 mV^2^/Hz × 10^–3^ vs. LA, *p* = .0216; and vs. LA + DZP, *p* < .001). However, none of the treatments was able to normalize the spectral power of the brainwaves (control: 0.50 ± 0.23 mV^2^/Hz × 10^–3^ vs. LA + DZP, *p* = .0473; and vs. both LA + PBT and LA + PHT, *p* < .001).

The decomposition of the brainwaves relevant to LA toxicity was also analyzed. In the case of the delta waves, there was significant variation among treatments (*F*_(4,40)_ = 94.63; *p* < .0001; Figure 5b) and delta power increased in the animals treated with LA in comparison with the control group (control: 0.09 ± 0.01 mV^2^/Hz × 10^–3^ vs. LA: 1.21 ± 0.10 mV^2^/Hz × 10^–3^, *p* < .001). All the anticonvulsants reduced the delta wave amplitude induced by LA (LA + DZP: 0.17 ± 0.08 mV^2^/Hz × 10^–3^ and LA + PBT: 0.49 ± 0.17 mV^2^/Hz × 10^–3^ vs. LA, *p* < .001; LA + PHT: 0.98 ± 0.27 mV^2^/Hz × 10^–3^ vs. LA, *p* = .0250), but only DZP was able to reduce it to the basal level (vs. control, *p* = .8104). Phenytoin had the least effect on delta wave amplitude compared with the other two anticonvulsant drugs (vs. LA + DZP and LA + PBT, *p* < .001).

As the beta wave is related to the convulsive process, the changes in the beta wave oscillations following treatment with anticonvulsants were also analyzed. Here, all the groups were significantly different from one another (*F*_(4,40)_ = 319.1; *p* < .0001; Figure 5c). Although all the treatments decreased the beta wave amplitude induced by LA (LA + DZP: 0.19 ± 0.05 mV^2^/Hz × 10^–3^; LA + PBT: 0.43 ± 0.05 mV^2^/Hz × 10^–3^; LA + PHT: 0.64 ± 0.08 mV^2^/Hz × 10^–3^; vs. LA: 0.89 ± 0.08 mV^2^/Hz × 10^–3^; *p* < .001), none of the treatment was able to reduce the amplitude to the basal level (control: 0.015 ± 0.001 mV^2^/Hz × 10^–3^; vs. all treatments, *p* < .001). Diazepam was the most effective anticonvulsant drugs tested (vs. PBT and PHT treatments, *p* < .001), while PHT was the least effective in the reduction of the beta wave amplitude (vs. LA + PBT, *p* < .001).

As in the case of the delta waves, significant variation was found among treatments in the distribution of gamma waves (*F*_(4,40)_ = 315.5; *p* < .0001; Figure 5d) and the LA‐treated animals presented increased gamma power in comparison with the control group (control: 0.009 ± 0.001 mV^2^/Hz × 10^–3^ vs. LA: 0.16 ± 0.01 mV^2^/Hz × 10^–3^, *p* < .001). Here, all treatments reduced the LA‐induced gamma power (LA + DZP: 0.02 ± 0.005 mV^2^/Hz × 10^–3^; LA + PTB: 0.05 ± 0.01 mV^2^/Hz × 10^–3^; LA + PHT: 0.08 ± 0.02 mV^2^/Hz × 10^–3^; vs. LA, *p* < .001 for all treatments), although PHT was less effective than DZP or PBT (vs. LA + DZP and LA + PBT, *p* < .001). No difference was found between the DZP treatment group and the control group (*p* = .0797).

### 3.4 Lidocaine‐induced seizures alter muscle contraction and are attenuated by anticonvulsant drugs

**FIGURE 6:**
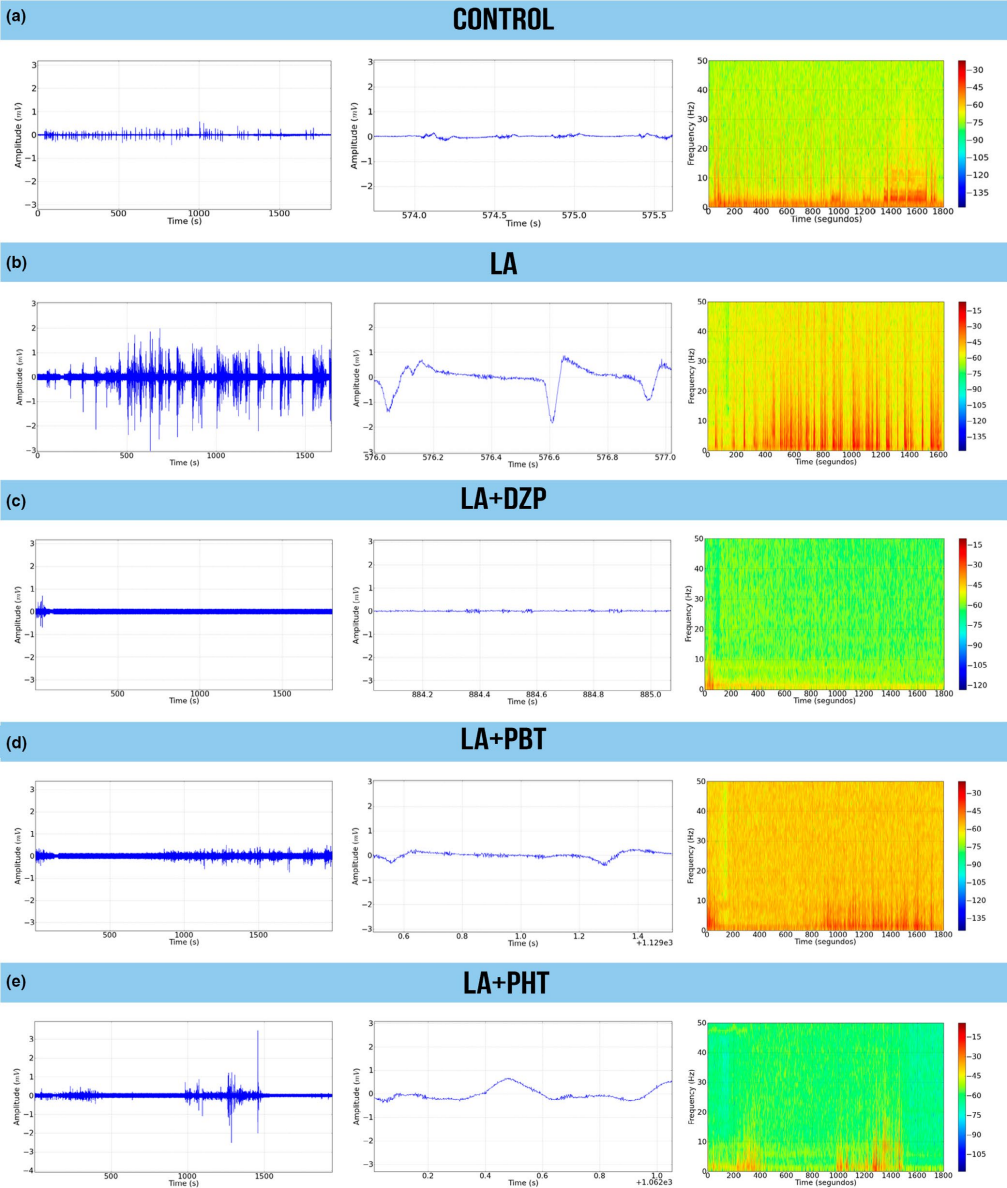
Surface electromyographic recordings of lidocaine‐induced seizures treated with anticonvulsant drugs performed for 30 min: Electromyographic recording of linear tracing performed by electrocorticographic (left); representative fragment of two seconds (center); and frequency spectrogram (right). (a) Control group. (b) Lidocaine group. LA‐induced muscle contractions treated by (c) diazepam, (d) phenobarbital, and (e) phenytoin. DZP, diazepam; LA, lidocaine; PBT, phenobarbital; PHT, phenytoin

Tonic–clonic seizures are associated with intense muscle contractions, which were measured here by EMG. The control EMG and the oscillations in its amplitude were measured in the masseter muscle (Figure 6a). In the LA group, intense muscle contractions were observed, and the EMG recorded oscillations in amplitude of up to 2 mV (Figure 6b).

In the DZP treatment, mild muscle contractions were observed at the beginning of the LA‐induced seizures, with amplitude of approximately 0.8 mV. The muscle contractions subsequently decreased to below basal level, reflecting the muscle relaxant proprieties of the DZP (Figure 6c).

The trace of the PBT indicates that muscle contractions were maintained at mild to moderate levels, but with reduced amplitude in comparison with LA alone (Figure 6d). However, greater excitability was observed in the PHT group, with reduced control of the muscle contractions (Figure 6e).

**FIGURE 7:**
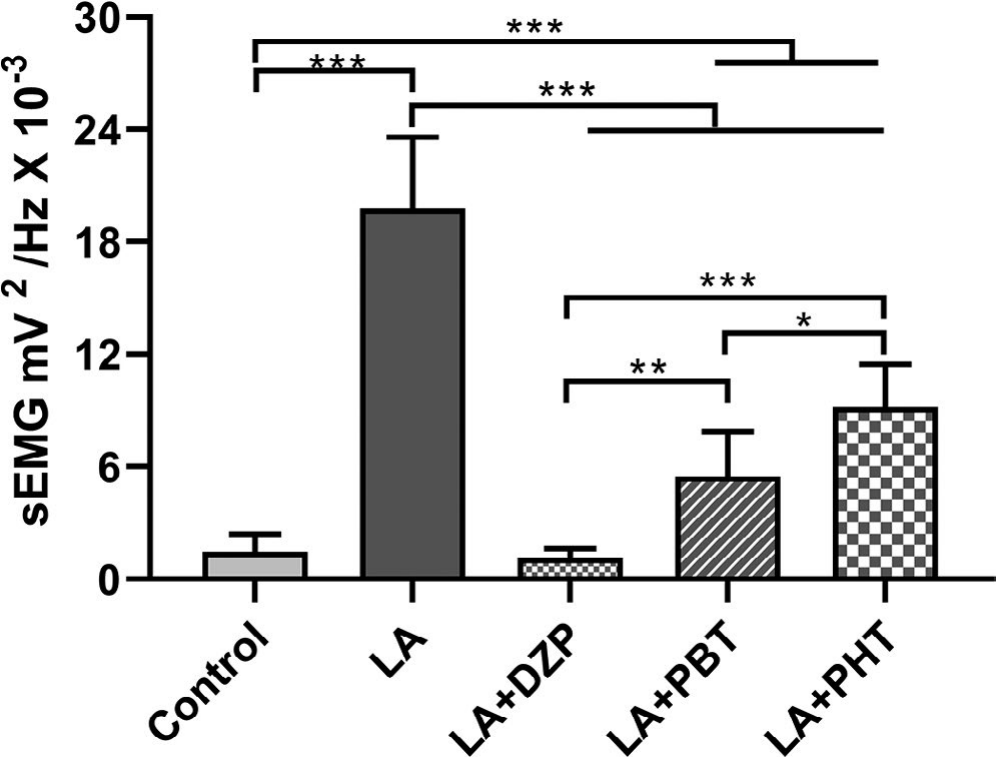
Quantitative linear distribution of the muscle contraction power in lidocaine‐induced animals and those treated with anticonvulsant drugs. The data are expressed as the mean ± *SD*(*n* = 9 per group; *p < .05; **p < .001; and ***p < .0001). DZP, diazepam; Hz, Hertz; LA, lidocaine; mV, millivolts; PBT, phenobarbital; PHT, phenytoin; sEMG, surface electromyography

The decomposition of the muscle contraction power distribution showed significant variation among treatments (*F*_(4,40)_ = 99.99; *p* < .0001; Figure 7). All treatments reduced significantly the muscle contraction induced by the LA (LA + DZP: 1.13 ± 0.51 mV^2^/Hz × 10^–3^; LA + PBT: 5.48 ± 2.41 mV^2^/Hz × 10^–3^; LA + PHT: 9.18 ± 2.28 mV^2^/Hz × 10^–3^; vs. LA: 19.80 ± 3.78 mV^2^/Hz × 10^–3^; *p* < .001 for all comparisons). Diazepam was the most effective treatment, reducing the muscle contraction power to levels similar to those of the control group (vs. control: 1.45 ± 0.96 mV^2^/Hz × 10^–3^, *p* = .9983; vs. LA + PBT, *p* = .0023; and LA + PHT, *p* < .001), whereas PHT was the least effective treatment (vs. control, *p* < .001; vs. LA + PBT, *p* = .0123).

## 4 DISCUSSION

The clinical use of LA as an anesthetic is a common and routine practice in medical and dental treatments (Lemming et al., 2019; Nagendrababu et al., 2019). However, there is a high rate of poisoning by LA, especially when it is not associated with a vasoconstrictor (usually adrenaline). Interestingly, parenteral formulations have the highest rates of accidental or intentional poisoning, as in intravenous anesthesia techniques (Centini et al., 2007; Kudo et al., 2004). The clinical signs of LA poisoning are observed by almost 10 min after administration, although the kinetics are poorly understood (Donald, 2004; Shannon et al., 2007).

Almost all types of local anesthetic that contain LA are toxic to nerve cells. This is why the initial symptoms and signs of toxicity caused by local anesthetics are usually neurological, ranging from numbness of the mouth to seizures (Hoffman et al., 2007). This is consistent with the findings of the present study, given the occurrence of seizure‐like behavioral changes in the rodents intoxicated with LA.

There is increasing evidence, with many case reports, of seizures induced by LA (Rahimi et al., 2018). This may occur even when administered by healthcare professionals (Alsukhni et al., 2016; Nicholas & Thornton, 2016), although major contributors include the indiscriminate use of local anesthetics and over‐the‐counter purchases (Aminiahidashti et al., 2015; Hoda et al., 2016), and even suicide attempts (Rahimi et al., 2018; Szadkowski et al., 2017).

Despite the existence of many reports of seizures induced by LA, little is known about this phenomenon. In the present study, LA was shown to be capable of inducing tonic–clonic seizures in rats which are consistent with the seizures described in a number of published case reports (Alsukhni et al., 2016; Aminiahidashti et al., 2015; Nicholas & Thornton, 2016). Records of brain activity during LA poisoning and responses to anticonvulsants are rarely found in the literature.

There are many types of seizure, which are characterized by different electroencephalographic patterns and behavioral correlates (Ding et al., 2019; Seneviratne et al., 2017). The LA‐induced seizure patterns described in the present study were characterized by moderate neuronal hyperexcitability, although this was less intense than that induced by PTZ, a positive control model for seizures. Although the total linear distribution of spectral power was greater in PTZ than the LA, the delta and gamma power was greater in LA than in the PTZ group, with its increase being related to tonic–clonic seizures (Binnie & Stefan, 1999). The underlying mechanisms of seizures caused by PTZ are due to antagonization of the GABA‐A receptor, which is characterized by cyclic neuronal recruitment firing. The breakdown of cerebral homeostasis by LA has a different trigger with the presence of isolated firing, with neuronal recruitment slower than PTZ.

Clearly, then, an increase in rapid brainwave activity is commonly described in neocortical seizures, both temporal and extratemporal (Lee et al., 2000; Perucca et al., 2014; Singh et al., 2015). From this perspective, the present study demonstrated an increase in the power of all the waves in comparison with the control, an increase in the power of the delta and gamma waves, and a decrease in the power of the beta waves in LA‐induced seizures, in comparison with the positive control. Previous studies have demonstrated that mesial temporal seizures are associated with fast activity in the beta and gamma frequency range (Bartolomei et al., 2004), whereas in extratemporal seizures there is an association only with the gamma frequency range (Lee et al., 2000; Singh et al., 2015). The findings of the present study are consistent with those of previous research, by showing that LA‐induced seizures present a mesial temporal seizure focus.

Lidocaine has been found to alter the concentration of intracellular Cl^−^ ions by attenuating the K^+^/Cl^−^ cotransporter, which extrudes Cl^−^ ions. This change leads to the inhibition of outward GABA‐induced currents, with a depolarizing shift of the GABA reversal potential (EGABA) and induced output currents, which reduce inhibitory GABA‐ergic activity (Nakahata et al., 2010). The inversion of the GABA receptor into EGABA excites GABA signaling due to chloride efflux (Zhu et al., 2014).

Based on evidence‐based guideline (Glauser et al., 2016), a benzodiazepine (DZP), a barbiturate (PBT), and an antiepileptic (PHT) were used here to control LA‐induced seizures. The results show that only DZP was able to reduce LA‐induced ECoG anomalies, although the total power distribution was reduced in all treatment in comparison with the LA group. Diazepam provided the best results, restoring delta and gamma wave power to baseline values and reducing beta wave power. PHT was the least effective anticonvulsant drug for the control of LA‐induced seizures. Based on the mechanism of action of the anticonvulsants, these data indicate that LA‐induced seizures may be involved with GABA‐mediated signaling. Due to DZP, better controlling *status epilepticus* induced by LA.

The results of the present study indicate that the animals treated with DZP had seizure activity of reduced duration and faster attenuation in comparison with the rats treated with PBT and PHT. The DZP treatment rapidly attenuated the amplitude of the trace after 15 min, corroborating the studies of Blas‐Valdivia et al. (2007) and Piña‐Crespo & Daló (2006). Jalilifar et al. (2016) found that the behavioral progression of the seizure is associated with an increase in delta oscillations. Overall, then, the present study showed a reversible increase in the delta and gamma bands in the rats treated with DZP, but not in those treated with PBT or PHT.

In addition to the ECoG data, LA‐induced seizures are also accompanied by intense muscle contraction. Given this, DZP was the only anticonvulsant capable of recovering LA‐induced muscle contractions to baseline levels. These findings are supported strongly by the muscle relaxant proprieties of DZP, which blocks muscle contractions by abolishing the polysynaptic reflex in the supraspinal centers and spinal interneurons, but without changing the excitability of the alpha motoneurons (Crestani et al., 2001). This muscle relaxant action occurs in the presence of physiological amount of GABA, given that the release of this neurotransmitter at the terminals of the primary afferent neuron will block the release of acetylcholine, and prevent excitatory stimulation (Crestani et al., 2001). The sum of these findings indicates that DZP is the most effective anticonvulsant for the control of seizure induced by LA, given its influence on brainwaves and surface electromyography.

## 5 CONCLUSION

Present study provided a detailed description of the ECoG features of the rat cortex during LA‐induced seizures. The use of LA provokes clear changes in the ECoG, which are characteristic of epileptiform activity. These alterations are suggestive of temporal zone seizures. Overall, the results of the study indicate clearly that DZP is the most effective of the three drugs used to control LA‐induced *status epilepticus*, in particular through the control and reduction of the delta and gamma waves. These findings indicate a potentially effective clinical treatment of the seizures induced by LA.

## CONFLICT OF INTEREST

The authors declare no conflict of interest.

## AUTHOR CONTRIBUTIONS

JFSS and LOF researched and analyzed the data and wrote the article. BGM, EJF, ASS, PSB, CAFM, and DACC researched and analyzed the data. VJM reviewed and edited the article. DCFL and MH designed and supervised the study and edited the article.

### Peer Review

The peer review history for this article is available at https://publons.com/publon/10.1002/brb3.1940.

## Data Availability

The data that support the findings of this study are available from the corresponding author upon reasonable request.
